# Effect of Heart rate on Basketball Three-Point Shot Accuracy

**DOI:** 10.3389/fphys.2018.00075

**Published:** 2018-02-06

**Authors:** Luca P. Ardigò, Goran Kuvacic, Antonio D. Iacono, Giacomo Dascanio, Johnny Padulo

**Affiliations:** ^1^Department of Neurosciences, Biomedicine and Movement Sciences, School of Exercise and Sport Science, University of Verona, Verona, Italy; ^2^Faculty of Kinesiology, University of Split, Split, Croatia; ^3^Wingate Institute, Zinman College of Physical Education and Sport Sciences, Netanya, Israel; ^4^Associazione Dilettantistica Basket Club 7 Laghi Gazzada Schianno, Gazzada Schianno, Italy; ^5^Sport Science, University eCampus, Novedrate, Italy

**Keywords:** fatigue, heart rate, task performance and analysis, sports, young

## Abstract

The three-point shot (3S) is a fundamental basketball skill used frequently during a game, and is often a main determinant of the final result. The aim of the study was to investigate the effect of different metabolic conditions, in terms of heart rates, on 3S accuracy (3S%) in 24 male (Under 17) basketball players (age 16.3 ± 0.6 yrs). 3S performance was specifically investigated at different heart rates. All sessions consisted of 10 consecutive 3Ss from five different significant field spots just beyond the FIBA three-point line, i.e., about 7 m from the basket (two counter-clockwise “laps”) at different heart rates: rest (0HR), after warm-up (50%HRMAX [50HR]), and heart rate corresponding to 80% of its maximum value (80%HRMAX [80HR]). We found that 50HR does not significantly decrease 3S% (−15%, *P* = 0.255), while 80HR significantly does when compared to 0HR (−28%, *P* = 0.007). Given that 50HR does not decrease 3S% compared to 0HR, we believe that no preliminary warm-up is needed before entering a game in order to specifically achieve a high 3S%. Furthermore, 3S training should be performed in conditions of moderate-to-high fatigued state so that a high 3S% can be maintained during game-play.

## Introduction

Due to its mixed physical-technical-tactical nature, basketball practice, like in other team sports, requires great attention to profiling the relevant physical and physiological characteristics of elite basketball players and, contextually, to determining the relationships between all of its features (e.g., metabolic demands) and the required technical skills. By investigating the typical game-driven physiological responses, recent research has widely determined the on-court activity patterns and the position-specific physical demands of this sport (Nikolaidis et al., 2014, 2015). In this regard, a body of studies has provided construct validity and reliability of physical assessment tests designed according to the running profile and activity patterns of the basketball players, by the inclusion of single or multiple changes of direction (Padulo et al., 2015b). Given the multi-faceted nature of basketball, whose performance success is a matter of physical, technical, and tactical ability, studies have been carried out as well regarding technical skills, such as the shooting task (Padulo et al., 2015a). A recent review (Okazaki et al., 2015) has reported that the ability to shoot an effective jump shot is critical for the player's success. Other findings are that players shoot more frequently in low-pressure and streaky situations (Csapo et al., 2015), and that compared to amateur players, professionals are able to shoot from greater distances and use more collective actions to find a shot position in which, possibly, the defensive pressure is lower (Ibáñez et al., 2009). Differences by playing position have been identified in short performers: point guards and power forwards shoot the most often and with the best accuracy in free-throw and two-point shots, whereas point guards shoot the most often and with the best accuracy in three-point shots (Ortega et al., 2006).

The above mentioned studies on factors influencing shooting performance have improved our understanding about the key factors targeted as fundamental for shooting performance accuracy. However, less information is available on the effect of fatigue, i.e., the variation of shooting accuracy over different exercise intensities, on the three-point shot (3S). In sport science, fatigue effects received attention, mainly because fatigue impacts overall athletes' performance (Faria et al., 2005). Harmful fatigue effects can diminish the function of a single whole-muscle, which leads to reduced muscle performance and therefore can decrease overall athletes' competition efficacy (Knicker et al., 2011). Fatigue can be described as a complex mechanism that involves both central and peripheral nervous system together with muscles (i.e., motor units; Ahmed, 2013). Poor perceptions, decisions, reactions, and resultant movement strategies are more likely to happen when athlete is in a fatigued state because central processing mechanisms and peripheral responses are compromised (Borotikar et al., 2008). In particular, the decision-making, considered as a high cognitive process, is closely related to fatigue and it can be said that a relation does exist between intensity and duration of the physical activity and this cognitive function (Abd-Elfattah et al., 2015). Commonly identified as an outcome of intense physical activity, fatigue has also been considered as a subjective experience, which can be described as a “sensation” (St Clair Gibson et al., 2003). Each individual has different sensations of fatigue and their generation is largely independent on the real biological state of the athlete, because brain uses the symptoms of fatigue as key regulators to protect body from potential damages (Noakes, 2012). Moreover, fatigue affects muscle strength, coordination, fine motor control, and movement patterns (Enoka and Stuart, 1992). Basketball players have high capability to move quickly, jump, and bounce the ball coordinating lower and upper limb movements (Cortis et al., 2011) and to achieve efficient basketball performance it is important to understand body adaptations and compensations under acute fatigue. Basketball players must be able to effectively perform specific tasks under conditions of physical fatigue that occurs during different training and game-play intensities (Kamandulis et al., 2013). Specifically about shooting performance, Barbieri et al. (2017) investigated fatigue effect on free-throw accuracy. They administered players a shuttle running fatiguing protocol and found postural control impairment but no free-throw accuracy decrease. Yet, by administrating such a protocol, they did not aim at achieving any reasonable heart rate-witnessed warm-up (Garrett and Kirkendall, 2000) and/or actual play (McInnes et al., 1995) metabolic intensity values.

Three-point shot performance is one of the main win determinants in elite basketball. Keeping a high 3S percent accuracy (3S%), especially during final minutes of close games, shows to be a key to success. Just as a reference, in the 2015–2016 season (regular season and playoffs), the NBA champions Cleveland Cavaliers shot—contested by players from the opposing team—3Ss in the first and last 2 min of regular quarters, and overtimes with a 3S% of 38.4 and 32.5%, respectively^1^ 3S is a common fundamental shot, which can be performed both when the players just come off the bench and when they are fatigued by previous actions. Knowledge about the effects of exercise intensity on 3S% would be of great importance for both sport scientists and basketball practitioners (e.g., coaches and fitness trainers). Since no previous study has been performed on this topic (Padulo et al., 2015a), sport scientists could use any further information as reference data for future studies on a basketball task performance and analysis model. In addition, coaches and fitness trainers might benefit from such knowledge in order to develop suitable exercise interventions for optimizing shooting accuracy. Therefore, the aim of the present study was to examine the effect of different heart rates on successful 3S%. We hypothesized that increasing heart rate would pair with decreasing 3S%.

## Materials and methods

### Participants

Twenty-four young (Under 17) basketball players (age 16.3 ± 0.6 yrs, height 180 ± 6.1 cm, mass 65.7 ± 7.2 kg, BMI 18.3 ± 1.7 kg/m^−2^, training experience 8.7 ± 2.6 yrs) were recruited from Associazione Dilettantistica Basket Club 7 Laghi Gazzada Schianno teams. All players, in addition do their weekly practice, participated in the seasonal championship made up of a regional phase, an inter-regional phase, and national 16-team finals. Inclusion criteria to participate in the study were: (i) participation in at least 85% of the previous season training sessions, (ii) regularly participating in the previous competitive season, (iii) having a valid sport medical certification, and (iv) being healthy (no pain or injury) and clear of any drug consumption. Participants refrained from drinking alcohol or caffeine-containing beverages for 24 h, and did not eat for 3 h, prior to testing to reduce any interference on the experiment. Each participant completed all trials in the same time period of the testing days (during the pre-season) and under the same climate conditions [4–7 p.m., 23.2 ± 0.6°C temperature and 55.3 ± 1.8% relative humidity (i.e., day times and climate conditions similar to real game-play)], to eliminate any influence of circadian variation. All tests were performed on a regular indoor basketball court and the participants wore their official basketball uniforms. Participants gave their assent, and written consent was obtained from the participants' parents/guardians after being thoroughly informed about the purpose, benefits, and potential risks of the study, in conformity with the Code of Ethics of the World Medical Association (Declaration of Helsinki). The protocol and the methods applied in the study were approved by the Ethical Committee of the Faculty of Kinesiology, University of Split.

### Protocol

**Figure 1:**
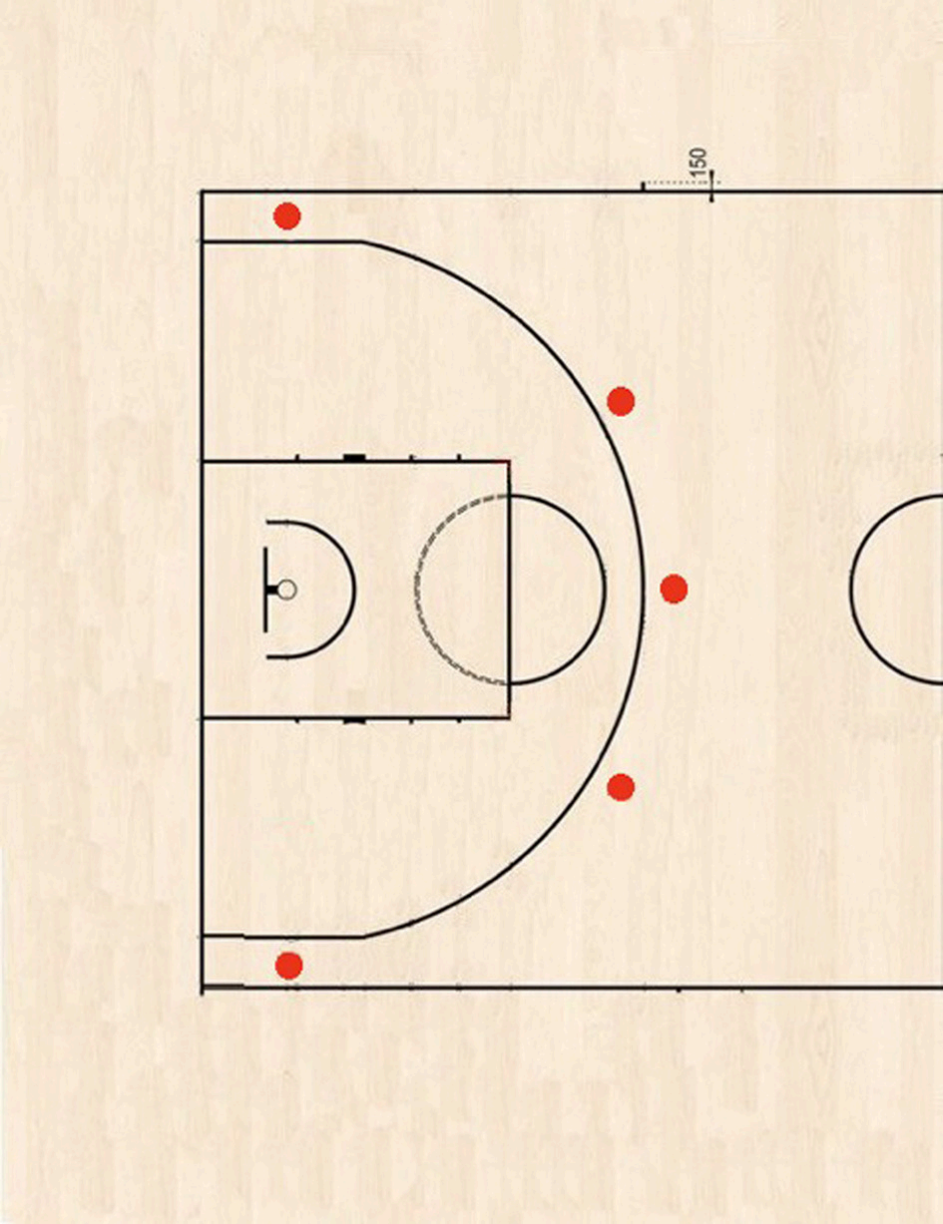
Three-point shot spots.

In the first session the participants performed a Yo-Yo Intermittent Recovery test level 1 (Yo-Yo IR1; Castagna et al., 2008b) to assess maximal heart rate (HRMAX). One week later the participants performed three randomized shooting testing sessions, with a 1-h rest plus warm-up between one session and the next one. All sessions consisted of 10 consecutive 3Ss (ball—Molten gf7, 600 gr.) from five different significant field spots just beyond the FIBA three-point line, i.e., about 7 m from the basket (similar to the NBA All-Star Weekend Three-Point Contest shots^2^; Figure 1, two counter clockwise “laps”) at three different heart rates (HR): rest (0HR), after warm-up with HR at 50%HRMAX (50HR; i.e., a reasonable post-warm-up HR value; Garrett and Kirkendall, 2000), and 80%HRMAX (80HR; i.e., a reasonable actual play HR value; McInnes et al., 1995), repeating the approach used by Padulo et al. (2015a). The same procedure was repeated 1 week later to evaluate the measures' reliability. More specifically, after a 15' standard warm-up run, each participant threw 10 consecutive 3Ss at different HRs (0HR—50HR—80HR). Each HR (continuously monitored with Cardio-Suunto™) was achieved by increasing the intensity of the shuttle running (15 + 15 m). Namely, the participants needed to run an average of 560 and 1600 m to achieve 50%HRMAX and 80%HRMAX, respectively (i.e., to elicit a post-warm-up- and actual play-like fatigued state).

### Statistical analysis

Measures of central tendency and dispersion (mean ± *SD* and SE) were computed from the Yo-Yo IR1 and 0HR, 50HR, and 80HR (effective %HRMAX and measured 3S%) test results to summarize the data. Statistical analyses were performed with SPSS 17.0 (SPSS Inc., Chicago, IL). Distribution normality of the population was tested with the Shapiro–Wilk test, and homogeneity of variances was verified with Bartlett's test. The reliability of the 0HR, 50HR, and 80HR tests was assessed by calculating the Intra-class Correlations Coefficient (ICC), according to the literature (Weir, 2005). Furthermore, a one-way within-participant's repeated-measures analysis of variance (ANOVA) was conducted to check for differences between the three test levels (0HR, 50HR, and 80HR) with a *post-hoc* Bonferroni test. Effect sizes are presented as partial eta-squared ($\eta_{\text{p}}^{2}$) to determine the meaningfulness of the results. Level of statistical significance was set at a *P* ≤ 0.05.

## Results

**Figure 2:**
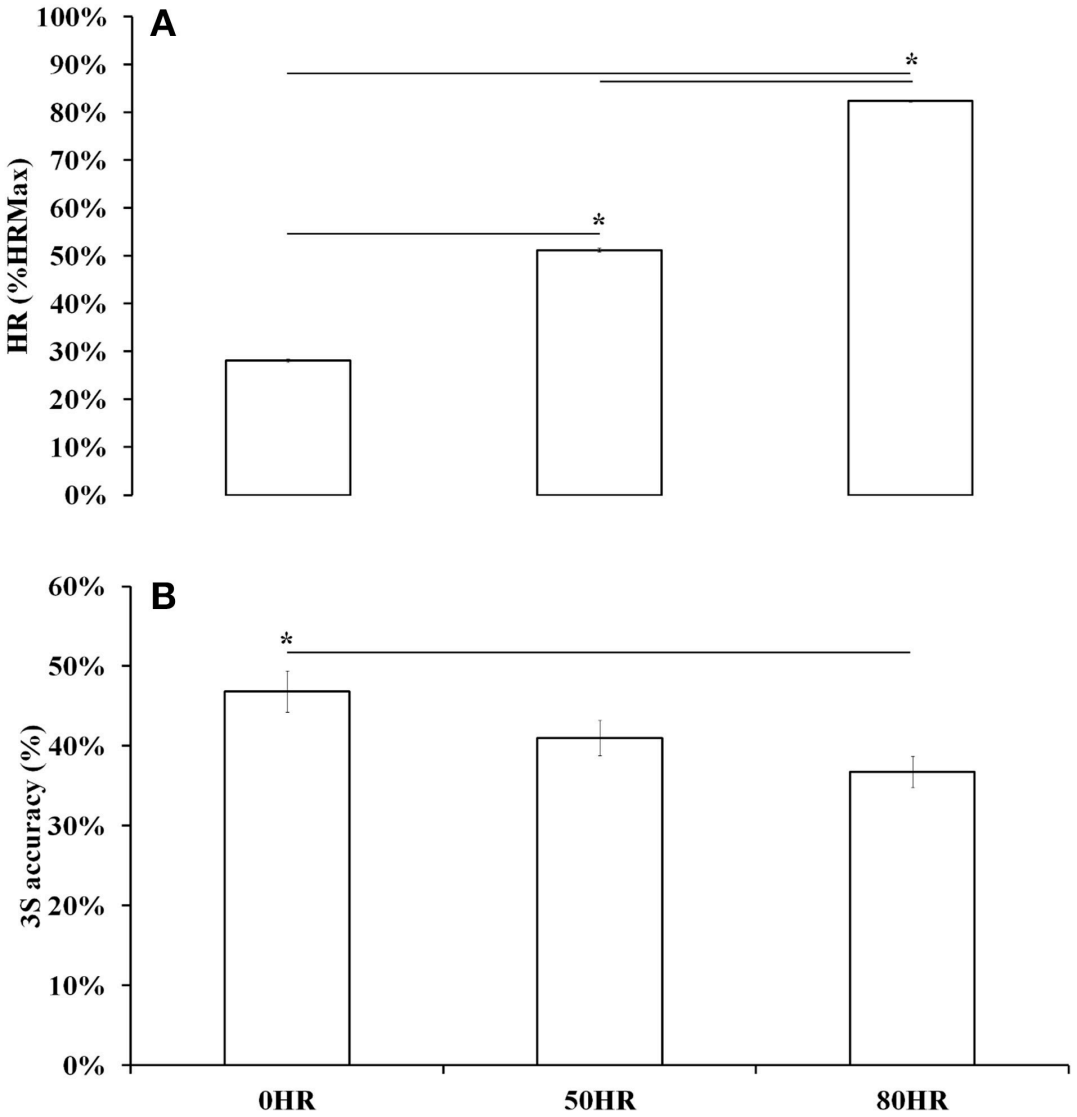
Heart rate [HR (%HRMax), **A**] and three-point shot percent accuracy [3S accuracy (%), **B**], as mean ± *SD*, over the three different testing conditions. ^*^P < 0.05.

ICC showed a good reliability at 0HR (0.88), 50HR (0.91), and 80HR (0.94). Yo-Yo IR1-derived HRMAX was 195.6 ± 6.1 bpm, obtained with a covered distance of 1,878 ± 568 m and a final speed of 18.4 ± 1.8 km/h. ANOVA confirmed differences of HR over the three administrated exercise conditions [Figure 2, top; *F*_(1.22)_ = 3405.722, *P* < 0.0001, η^2^_*p*_ = 0.990 at 0HR 54.4 ± 3.1 bpm, at 50HR 99.2 ± 4.6 bpm, and at 80HR 155 ± 6.5 bpm]. Similarly, ANOVA showed differences of 3S% over the three exercise conditions [Figure 2, bottom; *F*_(1.22)_ = 5.068, *P* = 0.009, η^2^_*p*_ = 0.131]. 3S% in the three exercise conditions was 46.8 ± 12.3% at 0HR, 41.3 ± 10.7% at 50HR, and 36.8 ± 9.5% at 80HR. The Bonferroni test did not show any significant 0HR−50HR 3S% (−15%, *P* = 0.255) or 50HR−80HR (−12%, *P* = 0.255) differences, whereas 80HR elicited significantly lower values of 3S% compared to 0HR (−28%, *P* = 0.007).

## Discussion

The major question of this study was whether there is any effect of different heart rates on successful three-point shot (3S) percentage (3S%). Therefore, three different heart rates were elicited to investigate 3S% in young male basketball players: rest (0HR), after warm-up with heart rate (HR) at 50%HRMAX (50HR), and 80%HRMAX (80HR). According to the results, the main findings of the present study can be summarized as follows: (i) 50HR does not significantly decrease 3S%, while on the other hand (ii) 80HR (i.e., intensity similar to that in real game-play; Ben Abdelkrim et al., 2010) significantly decreases 3S%. The difference in 3S% between 0HR and 50HR was about −15%, whereas the difference between 0HR and 80HR was (significantly) −28%. The adverse effects of fatigue on performance and skills in basketball (Lyons et al., 2006; Ahmed, 2013; Padulo et al., 2015a) have been reported in studies on other sports, such as tennis (Rota et al., 2014), water polo (Royal et al., 2006), and soccer (Russell et al., 2011). The results obtained by Padulo et al. (2015a) on the effect of fatigue on successful basketball free-throw (FT) percentage (FT%) are similar to the results of this study. Padulo et al. (2015a) reported that fatigue caused a decrease in FT% from −22% (between 80HR and 50HR) to −23% (between 80HR and 0HR). Both FT and 3S are complex basketball fundamentals, but are ecologically performed under very different game-play conditions. The FT is characterized by the fact that it is uncontested, which means that the shooter can be disturbed in a limited manner only by the opposing fans' movements and shouts (Padulo et al., 2015a). FT, which is a penalty for a player or team foul, contributes to from 15 to 21% of the total points scored during the game^3^ As a predominantly anaerobic sport, most of the energy demand for basketball's high-intensity activities, such as changes of direction, jumps, and shots, comes from the creatine phosphate system (CP; Metaxas et al., 2009). Yet a high fraction of maximum oxygen consumption is needed for recovery from such high-intensity activities (Tomlin and Wenger, 2001); it is therefore crucial for a player to recover in order to shoot successful FTs. A high level of aerobic fitness allows players to make a quick recovery after high-intensity activities, since muscle CP stores may be replenished within 30–40 s (Castagna et al., 2008a). It should be noted that in practice, a player is usually allowed about 30 s to shoot each FT (Padulo et al., 2015a).

In contrast, the 3S shooter must complete a motor action that is far more complex in order to achieve his successful task, i.e., managing the related decision making under fatigue, in a very small time frame (often with the approach of the shot-clock end), and with the opposition of one or more defenders (Oudejans et al., 2005). In addition, potential off-court disturbance, such as visual distractions, can slightly change FT's kinematics but without decreasing its accuracy (Viggiano et al., 2014). Two- (2S) and three-point shots provide the most points contributions during a match, with total score percentages of 51–67%^4^ and 16–35%^5^, respectively. 2S and 3S shots are the results of rapidly unfolding attack game plans, which very often include high-intensity movements. Given that the 3S requires more coordination and strength, elite basketball players can incur potentially more negative effects due to fatigue than in sub-maximal performances requiring relatively less overall effort such as FT (Uygur et al., 2010). In basketball, shot performance requires a highly coordinated entire body, from feet to hands, and this is especially true for long-range shots and 3Ss^6^ (Okazaki and Rodacki, 2012). Explosive movements, featuring fundamentals such as the jump shot and 3S, make fatigue effects on shooting accuracy even more relevant. Three-point shot accuracy decreases likely due to different reasons. We investigated its change (i.e., decrease) over changing metabolic demand (i.e., heart rate increase). What we found is at least a statistically significant link between the two signals. Like Padulo et al. (2015a) and Erculj and Supej (2009), we conclude that the training of shooting—be it FT or 3S—should be performed in conditions of from moderate-to-high fatigued state as well, so that an appropriate shooting technique can be preserved and will result in higher shooting accuracy during game-play.

## Conclusions

As a practical implication for coaches and players, given that 50HR does not significantly decrease 3S% with respect to 0HR, no preliminary warm-up is needed by the players before entering the game. In addition, since 80HR significantly decreases 3S% with respect to 50HR, it has come to light that 3S training in conditions of from moderate-to-high fatigued state is necessary to maintain high 3S% during game-play. The results of this study should prompt future studies on the effects of fatigued state on shooting accuracy—not limited to 3S but also on further (coordination-driven!) fundamentals such as defense ones—that administers fatiguing protocols more ecological than basic shuttle running, e.g., real attack game plans. It would be interesting to use alternative metabolic intensity proxies, such as Karvonen's HR reserve (Karvonen et al., 1957) and/or rate of perceived exertion (Borg, 1982), as well. In terms of study limitations, it should be considered with caution that results were obtained under very controlled conditions, while real game-play takes place in a sometimes chaotic setting characterized by opponents of varied levels, numerous score differences, opposing fans' behavior, etc.). Basketball statistics have provided indications about other confounding factors as well, such as shooters shooting better when defended and shooters scoring several points over a stretch of time without any special reason^7^ (i.e., the “hot-hand phenomenon or fallacy”, “streaky” shooters, and/or the “Matthew effect”; Gilovich et al., 1985; Wardrop, 1995; Koehler and Conley, 2003; Arkes, 2010; Csapo and Raab, 2014; Csapo et al., 2015). Another study limitation, that could prompt further studies, is that we did not considered heart rate effect on 3S% in different playing positions and/nor elite adult players (we chose to investigate a typical whole youth team). In conclusion, different metabolic conditions also affect a relevant basketball fundamental such as 3S, and coaches and trainers should consider this when designing effective specific training regimes.

## Author contributions

All authors listed have made a substantial, direct, and intellectual contribution to the work, and approved it for publication.

### Conflict of interest statement

The authors declare that the research was conducted in the absence of any commercial or financial relationships that could be construed as a potential conflict of interest.

## Footnotes

^1^BASKETBALL REFERENCE, http://www.basketball-reference.com/play-index/plus/shot_finder.cgi

^2^NBA ALL STAR, http://www.nba-allstar.com/contests/3point/

^3^2015-16 NBA season, TEAMRANKINGS, https://www.teamrankings.com/nba/stat/percent-of-points-from-free-throws?date=2016-06-20.

^4^2015-16 NBA season, TEAMRANKINGS, https://www.teamrankings.com/nba/stat/percent-of-points-from-2-pointers?date=2016-06-20

^5^2015-16 NBA season, TEAMRANKINGS, https://www.teamrankings.com/nba/stat/percent-of-points-from-3-pointers?date=2016-06-20

^6^BASKETBALL REFERENCE, http://www.basketball-reference.com/play-index/plus/shot_finder.cgi

^7^NBA, http://stats.nba.com
